# The Case for Special Hospitals

**Published:** 1888-12-01

**Authors:** Lennox Browne, Henry Power


### The Case for Special Hospitals.

By Mr. Lennox Browne, F.R.C.S. Edin., and Mr. Henry Power, F.R.C.S,

In the course of the discussion following the paper, at St. Bartholomew's Hospital, Mr. Lennox Browne expressed the opinion that Sir Sydney Waterlow had treated the whole subject in a concise and masterly manner. Of course the matter had been treated from the point of view of the Treasurer of a great and magnificent hospital, and as a critic of many smaller and more struggling institutions. No one, however, could complain of the way in which Sir Sydney had criticised that with which he did not entirely agree. As to small hospitals, there were small hospitals and small hospitals, as the Distribution Committee of the Hospital Sunday Fund well knew; but he maintained that there were some small hospitals which did their work as well and as economically—with their means—as the large hospitals, while he did not feel sure that the work of the large hospitals was always done as economically as it was claimed they did. Speaking as a specialist, he considered that the truism which had been quoted from across the Atlantic that "Legitimate specialism should be regarded only as a superstructure built on a substantial foundation of generalism," was neither very original nor very profound. But where, he asked, were the students to get their special instruction? Clearly not till they had passed through their general curriculum, and until the examining boards made it a feature to test students on special questions, or until honours were given to men who showed proficiency in special branches at qualifying boards other than the London University and some of the institutions where higher degrees were obtained, there would be no motive for the general mass of students to take up special subjects. Of course, the subjects of general surgery and general medicine were much more serious matters to study now than they used to be, and specialists were being developed because of the necessity for subdividing these general branches, on account of these very innovations and improvements. It was a mistake to say that there were no pupils or classes at special hospitals; they were to be found at all special hospitals of any reputation, although schools and large fees were absent, and these students came from among the most distinguished of the general hospitals. At the hospital with which he was connected he had the honour and the advantage of the assistance of most distinguished students, even from that great hospital, and there were many who were Fellows of the Royal College of Surgeons by examination came to him to learn something which they had not always learnt in the special departments of the general hospitals to which they had been attached. Sir Sydney, in speaking of these special institutions, had said he admitted their necessity for contagious diseases, lying-in, or consumption. He (the speaker) quite agreed in the advantage of separating the first two, but could not understand why consumption should always be claimed as a proper subject for a separate hospital. If there was anything in the modern view of consumption—and he for one believed that there was—he thought that the congregation of a large number of consumptive persons in one hospital, and especially in the metropolis, was by no means a good or advantageous thing for the patients. There were many other hosptials entirely sentimental, and the Cancer Hospital was one. No great scientific work had come out of that hospital, such as had occurred from the Middlesex Hospital, so honourably associated in its cancer wards with the names of Moore, Campbell de Morgan, and Nunn, and in the absence of result he held that this disease could be treated equally well, if not better, in a general hospital. The most cogent and justifiable reason for special hospitals was *technique*—that is, hospitals for diseases which required special methods and instruments for examination and treatment, involving protracted teaching and continued practice. He reminded the meeting that one of the greatest hospitals of this character in this metropolis —the Moorfields Ophthalmic Hospital—was treated with the most absolute ridicule, and was criticised most severely by the *Lancet*, until it made such a name by its magnificent work that there was now hardly a surgeon in this City with a knowledge of the diseases of the eye who had not come from Moorfields, whereas before its institution there was no ophthalmic department at any general hospital. This last remark applied equally to the throat, diseases of which organ constituted, he said, another specialty necessitating a knowledge of *technique*, and it was a curious fact, upon which he challenged contradiction, that the surgeons in charge of the special departments of the London Hospital, St. Bartholomew's, St. Thomas's, Westminster, St. George's, St. Mary's, and Charing Cross, were all indebted for their knowledge in these diseases to the Throat Hospital in Golden Square, with which he was not connected officially, though he gratefully acknowledged that the foundation of his knowledge of throat diseases was derived from constant study in that institution for a period of seven years. People did not quite know the work which special hospitals were doing, and that really many of the best students of the general hospitals went to them voluntarily to improve themselves in a way they found they were not always able to do at the general hospitals.

Mr. Henry Power said: Some of the special hospitals are rather hardly dealt with by the Distribution Committee of the Metropolitan Hospital Sunday Fund. He attributed the improvement which, he said, had obviously occurred in the character of the students, to the circumstance that the education, partly due to instruction received at special hospitals, was very much better, while the knowledge which was required of a young student nowadays was vastly superior to what it used to be. He considered, however, that students were required to learn too many facts from books, many of which were relatively unimportant. However, the result of the present system of education had been to introduce into the profession a large body of very highly-educated men, who were competent generally to do their work well in the world.

